# World Humanitarian Day: Why humanitarian settings need more research, not less

**DOI:** 10.1371/journal.pmed.1005220

**Published:** 2026-08-18

**Authors:** Akihiro Seita

**Affiliations:** Department of Health, United Nations Relief and Works Agency for Palestine Refugees in the Near East (UNRWA), Amman, Jordan

## Abstract

In this Editorial, Akihiro Seita outlines how and why medical journals should recognize that while conventional study designs are often not possible in humanitarian settings, this does not undermine the quality or rigor of such research, and outlines why it deserves greater support and recognition.

## Teaser

Humanitarian crises are among the defining global health challenges of our time, yet medical research from these settings remains underrepresented. Medical journals should recognize that while conventional study designs are often not possible in humanitarian settings, this does not undermine the quality or rigor of such research, which deserves greater support and recognition.

## Introduction

Every year on 19th August, the world marks World Humanitarian Day to honor humanitarian workers who continue to serve people affected by conflict, disasters, and displacement. It is also a reminder that hundreds of millions of people continue to suffer unnecessarily from humanitarian crises that threaten their health, dignity, and lives. Supporting humanitarian workers is thus a shared global responsibility, but it is often perceived as the responsibility of humanitarian organizations alone, rather than something to which everyone can contribute. I often receive questions from the medical community about how they can contribute to humanitarian work: there are of course many ways, but one important area of support is medical research.

This is primarily because of the universal core of medical research, which is to generate knowledge that improves people’s health and well-being, particularly for those who are most vulnerable. By identifying effective interventions and informing clinical practice and public health policy, research provides the evidence needed to improve humanitarian responses and save lives. This fundamental value of medical research does not vary from humanitarian to clinical to health systems research.

At the same time, medical research has specific roles in humanitarian crisis, including: ensuring that the suffering and needs of crisis-affected populations are documented, understood, and translated into action; helping to make health services more resilient; and contributing to health systems development once the conflict is over. One example is diabetes care. Research documenting the substantial burden of diabetes and the major barriers to care in humanitarian settings helped establish noncommunicable diseases as an essential component of humanitarian health responses, after years of being largely overlooked despite their considerable disease burden [1]. Subsequent research also addressed practical operational challenges, demonstrating that several insulin formulations remain stable under fluctuating tropical temperatures, providing evidence to support safer insulin use where reliable refrigeration is unavailable [2].

In humanitarian settings, the priority is always operational: to save lives and maintain essential health services. At the United Nations Relief and Works Agency for Palestine Refugees in the Near East (UNRWA) in Gaza, for example, our priority is to continue providing primary healthcare to people in need. Since the start of the war, UNRWA has provided more than 18 million medical consultations, making it the single largest provider of primary healthcare in Gaza [3].

Yet, humanitarian action is inherently complex. Every day, organizations such as UNRWA must decide how best to deploy limited staff, medicines, vaccines, and financial resources. These decisions are extraordinarily difficult because health threats, population movements, and humanitarian needs change rapidly, while there is often little evidence or relevant guidance to inform decision-making. Paradoxically, research may be needed even more in humanitarian settings than in stable environments.

We therefore need to support both the conduct and publication of medical research in humanitarian settings. Although humanitarian crises have become one of the defining health challenges of our time, research and perspectives from these settings remain relatively uncommon in flagship medical journals. This is understandable; conducting rigorous research during armed conflict is extraordinarily difficult, and conventional study designs are often impossible to implement. Protecting frontline workers and researchers must always remain the highest priority. Nevertheless, conducting meaningful research is not impossible. UNRWA’s experience in Gaza provides one such example.

In 2024, UNRWA began identifying malnourished children in Gaza and providing therapeutic feeding. To better understand the evolving nutritional crisis, UNRWA integrated nutrition surveillance into its routine primary healthcare services across Gaza. Through 16 health centers and 78 medical points, frontline health workers measured the mid-upper arm circumference of more than 220,000 children, generating over 265,000 measurements despite active conflict, repeated displacement, and the collapse of much of the health system. The surveillance system informed humanitarian responses in real time, helping to better understand the evolving nutritional situation and support operational decision-making. It also contributed to the international understanding of the nutritional crisis in Gaza and was subsequently published in *The Lancet* [4].

Three factors made this possible. First and foremost was the commitment of UNRWA’s frontline health workers in Gaza. More than 1,000 health staff working in health facilities continued to provide care to children while recording and reporting their nutritional status despite the ongoing conflict. Second was the presence of researchers at UNRWA headquarters in Jordan; working closely with colleagues in Gaza, they received, cleaned, analyzed, and interpreted the data. Data cleaning was particularly challenging because of the complexity of collecting data during an active conflict. Third was the partnership with the Johns Hopkins Bloomberg School of Public Health, whose researchers provided scientific guidance and overall research oversight of the study.

Research conducted in war settings can never be perfect, and neither was the study in Gaza [4]. Still, discussions with the publisher on how general medical journals should evaluate research conducted in humanitarian settings allowed consideration of the realities of conflict, displacement, and resource-limited environments that mean conventional study designs are often neither feasible nor, at times, ethical. This does not mean that standards of scientific rigor or research integrity should be lowered. Rather, journals should recognize that rigorous research may take different methodological forms depending on the context. A thoughtful appreciation of these constraints, while maintaining high expectations for quality, would help ensure that important evidence generated in humanitarian settings receives the visibility it deserves.

Ultimately, humanitarian settings should no longer be regarded as the periphery of medical research when they have become one of its defining frontiers. The better we understand humanitarian crises through research and reliable data, the better we can respond to them. At a time when humanitarian crises are becoming more frequent, more complex, and more prolonged, bringing evidence from these settings into mainstream medical journals is not simply a matter of representation; it is an essential investment in global health.
